# A Concise Review on Taro Mucilage: Extraction Techniques, Chemical Composition, Characterization, Applications, and Health Attributes

**DOI:** 10.3390/polym14061163

**Published:** 2022-03-15

**Authors:** Mansuri M. Tosif, Agnieszka Najda, Joanna Klepacka, Aarti Bains, Prince Chawla, Ankur Kumar, Minaxi Sharma, Kandi Sridhar, Surya Prakash Gautam, Ravinder Kaushik

**Affiliations:** 1 Department of Food Technology and Nutrition, Lovely Professional University, Phagwara 144411, Punjab, India; tosifmansuri444@gmail.com; 2 Department of Vegetable and Herbal Crops, University of Life Science in Lublin, Doświadczalna Street 51A, 20280 Lublin, Poland; 3 Department of Commodity Science and Food Analysis, Faculty of Food Science, University of Warmia and Mazury in Olsztyn, Oczapowskiego 2, 10719 Olsztyn, Poland; klepak@uwm.edu.pl; 4 Department of Biotechnology, CT Institute of Pharmaceutical Sciences, South Campus, Jalandhar 144020, Punjab, India; aarti05888@gmail.com (A.B.); gautamsuryaprakash@gmail.com (S.P.G.); 5 Central Instrumental Lab, National Institute of Food Technology Entrepreneurship and Management, Sec-56, Plot-97, Kundli, Sonipat 131028, Haryana, India; ankur_chem97@rediffmail.com; 6 Department of Applied Biology, University of Science & Technology, Techno City, Killing Road, Baridua 9th Mile 793101, Meghalaya, India; 7 UMR1253, Science et Technologie du Lait et de l’œuf, INRAE, L’Institut Agro, Agrocampus Ouest, 65 Rue de Saint Brieuc, F-35042 Rennes, France; sridhar4647@gmail.com; 8 School of Health Sciences, University of Petroleum and Energy Studies, Dehradun 248007, Uttrakhand, India; ravinder_foodtech2007@rediffmail.com

**Keywords:** mucilage, biopolymer, polysaccharide, food applications

## Abstract

Taro (*Colocasia esculenta*) is an important source of carbohydrates as an energy source and is used as a staple food throughout the world. It is rich in mucilage and starch granules, making it a highly digestible ingredient. Mucilage can act as a matrix and a thickening, binding, emulsifying, or foaming agent in food, pharmaceutical, and several other fields of research. Moreover, mucilage can be extracted from several living organisms and has excellent functional properties, such as water-holding, oil-holding, and swelling capacities. Therefore, these remarkable functional properties make mucilage a promising ingredient with possible industrial applications. Furthermore, several extraction techniques, including enzyme-assisted, ultrasonication, microwave-assisted, aquatic, and solvent extraction methods, are used to obtain quantitative amounts of taro mucilage. Coldwater extraction with ethanol precipitation can be considered an effective and cost-effective technique to obtain high-quality mucilage with suitable industrial applications, whereas the ultrasonication method is more expensive but results in a higher amount of mucilage than other emerging techniques. Mucilage can also be used as a fat replacer or reducer, dye remover, coating agent, and antioxidating agent. Therefore, in this review, we detail the key properties related to the extraction techniques, chemical composition, and characterization of taro mucilage, along with its suitable applications and health benefits.

## 1. Introduction

Over the past years, the adverse effects of synthetic polymers on human health and the environment (non-biodegradability, non-biocompatibility, and cyst formation) have limited their usage in therapeutic and industrial applications [1,2]. Therefore, interest in plant-derived viscous polysaccharides, including mucilage and gums, has been increasing in the food, pharmaceutical, and other industries due to their diverse functional properties, such as emulsifying, binding, coating, gelling, thickening, and stabilizing properties [3]. Furthermore, mucilage is an eco-friendly, cost-effective, edible polysaccharide and is mainly extracted from living organisms, e.g., bacteria, fungi, plants, animals, and algae [4]. Among the plant kingdom, flaxseed, taro, chia seed, psyllium, basil seed, tamarind seeds, and okra are the richest sources of mucilage. Moreover, mucilage has been exploited in the human diet (as a food ingredient substitute or additive) [5,6,7]. Even at low concentrations, mucilage has excellent viscosity, water-holding capacity, oil-holding capacity, and antimicrobial activity. Thus, these remarkable functional properties make mucilage a promising ingredient with possible applications as a fat replacer [8], gel former [9], thickener [10], and emulsifier [11]. Chemically, mucilage is a physiological product of plant metabolites and is composed of polysaccharide units of L-rhamnose, D-galactose, D-xylose, and L-arabinose. It also contains organic acids and a small number of proteins [12]. The physicochemical, functional, and chemical compositions of mucilage are highly dependent upon the origin and the types of extraction and purification methods [9]. For example, okra mucilage is composed of (1,2)-rhamnose and (1,4)-galacturonic acid residues with disaccharide side chains [13]. Similarly, psyllium mucilage is composed of β-D-1,4-linked xylo-pyranose components substituted by ά-L-arabino-furanose fractions with a xylose/arabinose ratio of 3:1 [14].

Taro (*Colocasia esculenta*), a plant of the Araceae family, is a very important staple crop in the human diet and is mainly grown in the humid tropical regions of the world. It has been reported that taro is a potential source of about 3 to 19% mucilage and 70 to 80% starch, depending upon the extraction method [15,16]. Moreover, the chemical composition of taro mucilage comprises carbohydrates (glucose, galactose, mannose, xylose, and arabinose) and proteins (major amino acids, e.g., leucine, isoleucine, cysteine, tryptophan, and lysine). The presence of radicals of completely or partially hydrophobic amino acids, such as tryptophan, isoleucine, and leucine, can improve the emulsifying properties of taro mucilage [17,18]. It also contains high amounts of arabinogalactan-proteins (AGPs). Thus, fractions of proteins with non-polar radicals present in taro mucilage are thought to be responsible for the hydrophobic portion, while carbohydrates contribute to the hydrophilic character. Furthermore, several reports on taro mucilage revealed that starch was a major impurity in mucilage that may affect its techno-economical applications [19,20].

Numerous reports have been published on the effect of taro mucilage on baked food products. For example, Travares et al. [21] used taro mucilage in sliced bread as a stabilizing or thickening agent, resulting in reduced fat levels in high-quality bread. Likewise, the emulsifying properties of taro mucilage were found to be beneficial for the quality and desirable production of bread in a study by Contado et al. [22]. Only a few reports have been published on taro health attributes and taro starch. However, no comprehensive review is available on taro mucilage. Therefore, this review provides comprehensive details about the extraction techniques, chemical composition, characterization, and health attributes of taro mucilage. Additionally, several applications of taro mucilage are discussed, along with mechanisms and schematic diagrams.

## 2. Extraction Process and Compositional Properties of Taro Mucilage

**Figure 1:**
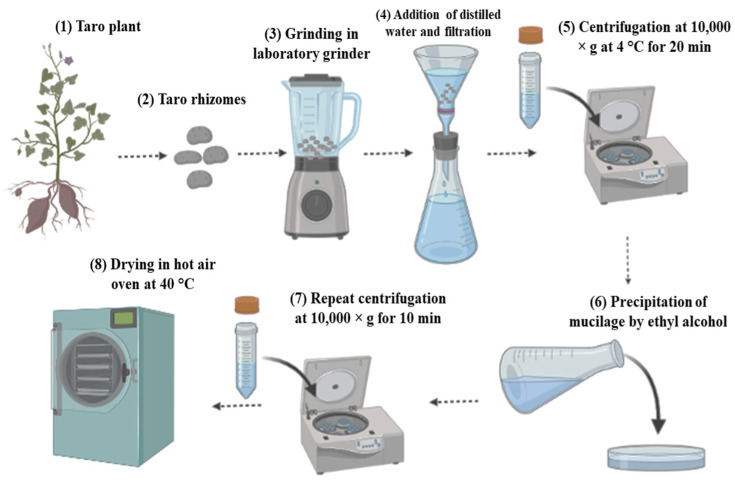
Schematic representation diagram of the extraction of taro mucilage at low temperature with ethanol precipitation.

Mucilage extracted from different plant parts has diverse applications and characteristics based on their respective functional and structural components. In addition, several extraction techniques, including enzyme-assisted, ultrasonication, microwave-assisted, aquatic, and solvent extraction methods, are used to obtain quantitative amounts of mucilage [23]. However, very few techniques have been adopted for the extraction of taro mucilage (Table 1). Centrifugation and drying techniques have been used in order to obtain dried powder or purified mucilage [24]. Additionally, it has been reported that the oven-drying technique is more effective than the spray dryer because it operates at a high temperature for a short time [25]. Starch present in taro mucilage can be considered a major impurity that affects its quality and is not beneficial for emulsification [26]. Apart from extraction methods, various factors such as maturity stage, distribution, molecular size, degree of branching, structural linkages, presence of hydrophobic components, and monomeric compositions also affect the functional properties of taro mucilage [27,28]. Similarly, the physiological stages of taro rhizomes showed variation in the yield of mucilage, as observed by Tavares et al. [21]. Higher starch content was noted six months after planting and was reduced at the eighth month, whereas a high amount of dry matter was found at the physiological maturity stage of taro, and high protein content was found before the maturity stage. Ultrasonication in combination with hydrodynamic forces is widely adopted in order to increase the mechanical potential of the highly viscous medium [29]. Consequently, Lin and Huang [30] extracted high-purity (98%) mucilage from taro corms using a low-temperature method. In this study, high-performance liquid chromatography (HPLC) was used to analyze the acid hydrolysates of mucilage, and D-galactose (61.6%) was determined to be a major component, followed by D-glucose (19.7%) and D-arabinose (16.2%). Similarly, in a study by Andrade et al., taro mucilage was extracted in five different conditions, i.e., (a) at room temperature, (b) at room temperature with ethanol precipitation, (c) at high temperature, (d) at high temperature with ethanol precipitation, and (e) at low temperature with ethanol precipitation [31]. Higher yield (8.05%) was obtained at room temperature, and the highest emulsifying activity and stability were found in condition (b), mucilage extracted at room temperature with ethanol precipitation. On the other hand, condition (d) at high temperature with ethanol precipitation and condition (e) at low temperature with ethanol precipitation was highly reflected in the precipitation of mucilage with ethanol due to the solubilization of non-starch short-chain carbohydrates and other components. In this regard, the extraction of mucilage at low temperature with ethanol precipitation is explained in Figure 1. In this process, after cleaning, peeling, and crushing, rhizomes are filtered by adding distilled water. Afterward, the filtered solution is centrifuged, the supernatant is collected, and mucilage is precipitated by ethyl alcohol. Moreover, acid- and alkali-extracted mucilage showed less viscosity and higher yield as compared to water-extracted mucilage due to compositional differences in monosaccharides in the mucilage structure [3].

**Figure 2:**
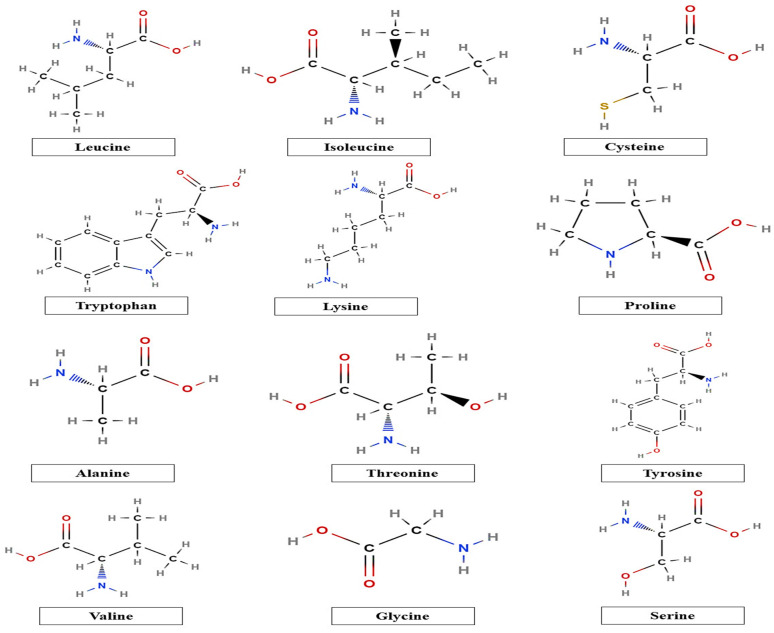
Chemical structures of different amino acids present in taro mucilage.

Furthermore, the chemical composition, including monosaccharide units and amino acids, and proximate composition of taro mucilage are also dependent upon the extraction techniques of mucilage. Taro mucilage consists of carbohydrates (arabinose, rhamnose, arabinose, fructose, mannose, galactose, fructose, and glucose) and also contains various amino acids, as shown in Figure 2. The high glucose content present in taro mucilage is due to the presence of starch during the extraction. In addition, taro mucilage is a rich source of high amounts of arabinogalactan-protein (AGP) (93–98%) and provides a high amount of AGP, which is responsible for the improvement of the emulsifying properties of mucilage due to the presence of hydrophilic and hydrophobic amino acids, as stated by Andrade et al. [32] and Njintang et al. [33]. Several phenolic compounds present in taro mucilage are esterified (bound phenolic compounds), which are interconnected to arabinose residues or carboxyl groups via ester bonds. These esterified compounds have great potential to inhibit enzymatic activity by polyphenol oxidases (PPO) such as laccase, which results in the improvement of functional properties.

Consequently, Njintang et al. [33] extracted mucilage from five different taro varieties by solvent extraction treatment. In their work, the protein content of mucilage was found to be higher (30–51%) due to the use of saline buffer during the extraction. Therefore, different growth conditions of taro can affect the protein content, carbohydrate proportion, and yield of mucilage. Similarly, taro mucilage also contains acidic glycoproteins, acidic glycans, and natural glycans. These fractions of glycans are considered effective viscosity modifiers and gelling agents according to Manhivi et al. [34]. Carbohydrates, ash, fiber, moisture, lipids, and proteins are major components constituting the proximate compositions of foods. However, several authors have obtained different results for the proximate compositions of taro mucilage [35]. For example, the chemical composition of mucilage extracted at low temperature with ethanol precipitation showed an average ash content, a lower amount of crude fiber and ethereal extract, and a higher amount of the glycosidic fraction and protein content in the inorganic fraction of mucilage [31], whereas mucilage extracted at room temperature without precipitation showed a decreasing trend of glycosidic fractions from 91% to 38%, and protein content was increased from 3% to 47%. These findings were caused by the elimination of carbohydrates, including starch components, after centrifugation in the cold treatment [32]. Moreover, several reports have revealed that bonding between proteins and carbohydrates is responsible for the emulsifying properties of mucilage [36].

## 3. Characterization of Taro Mucilage

### 3.1. Fourier Transform Infrared Spectroscopy

Fourier transform infrared spectroscopy (FTIR) is an analytical method used to evaluate the chemical bonds or functional groups existing in mucilage or any other molecule by producing an infrared absorption spectrum [39]. Several authors have documented that taro mucilage is constituted by C=O, CH_3_, OH, C-O, CH, and C-O-H groups. For instance, axial deformation of the CH bond was assigned to bands at 2955, 2934, 2916, 2943, 2935, and 2907 cm^−1^. The presence of the C=O stretching of peptide groups was confirmed by bands at 1700 and 1600 cm^−1^, which is known as the amide I band. Therefore, these FTIR results revealed the presence of protein fractions in taro mucilage, as proven by Andrade et al. [31]. In another study, axial deformation of a hydroxyl group (-OH) (at 3300 and 3200 cm^−1^) and a C-H bond (at 3000 and 2840 cm^−1^) was found, which confirms the presence of carbohydrates in taro mucilage. Moisture and N-H bonds in proteins are responsible for amide I, amide II, and nucleic acids attributed to the region between 1750 and 1500 cm^−1^, according to the study of Andrade et al. [32]. Chawla et al. [4] reported that the spectral fingerprint for AGP glycoprotein or carbohydrate is the region between 1200 and 800 cm^−1^. Consequently, Manhivi et al. [34] investigated the functional groups of mucilage extracted from taro and cactus. For taro mucilage, axial deformation of the hydroxyl group (-OH) was found with intramolecular bonding in the range between 3000 and 3600 cm^−1^. However, C-O stretching of carboxylic acids was confirmed between 1320 and 1210 cm^−1^ for mucilage from both sources.

### 3.2. Scanning Electron Microscopy

Scanning electron microscopy (SEM) is used to characterize the microstructure of the surface morphology of the polymer. Starch is a part of mucilage, and the gelatinization of starch occurs at a high temperature, which results in the swelling of starch granules [40]. This phenomenon is characteristic of starch granules in a suspension at a high temperature. However, non-gelatinized starch was found in the case of mucilage extracted at high temperature with ethanol precipitation, as confirmed by SEM by Andrade et al. [31]. The surface morphology of taro mucilage incorporated into hydroxypropyl methylcellulose (HPMC)-based transdermal patches were characterized by SEM by Sarkar et al. for further application in drug delivery [38]. The results of SEM revealed that the surface morphology of drug-loaded pure HPMC patches was smooth and uniform, whereas mucilage patches showed a unique interconnected network structure. On the other hand, the patches retained the skeletal backbone for drug release, which indicates that drug release is highly diffusion controlled. Therefore, it is possible that a combination of mucilage and HPMC could be an effective agent for sustained release in a transdermal drug delivery system. Similarly, grafted polylactide copolymers with taro mucilage were produced using conventional approaches and microwave irradiation and then visualized under different magnifications [41]. The resulting images of mucilage morphology indicated a rough surface and irregular granular shapes. However, mucilage grafted with lactide appeared whiter and rougher, but the morphology reformed from a granular structure to a flat structure after the grafting of lactide chains onto its backbone.

### 3.3. Differential Scanning Calorimetry and Thermal Gravimetric Analysis

Differential scanning calorimetry (DSC) is a technique to investigate how polymers respond to heat. The melting of a crystalline polymer or the glass transition can be studied using DSC [42]. The difference in the amount of heat required to raise the temperature of a sample compared to a reference is assessed as a function of temperature in the DSC technique. Mijinyawa et al. [41] studied the DSC of taro mucilage and grafted polymer (polylactide copolymers with taro mucilage). In comparison to grafted copolymers, taro mucilage had higher-intensity crystalline diffraction peaks due to its high crystallinity. Furthermore, in semi-crystalline polymers, the destruction of the hydrogen bonding between the chains can result in a reduction in XRD peak intensity and therefore reduced crystallinity. Similarly, the DSC thermal profile of taro mucilage revealed a broad endothermic transition with an onset temperature of 44 °C and a peak temperature of 111 °C because mucilage contains heterogeneous hydrophilic carbohydrates that contain both crystalline and amorphous polysaccharides. Since degradation is exothermic, there was no evidence of depolymerization of taro mucilage up to 195 °C, as reported by Manhivi et al. [34].

Thermal gravimetric analysis (TGA) is a type of thermal analysis in which the mass of a sample is measured as temperature varies over time. This measurement provides information about physical parameters such as desorption, adsorption, absorption, and phase transitions [42,43] and chemical parameters such as thermal decomposition, chemisorption, and solid-gas reactions. A thermogravimetric analyzer continually measures the mass while the temperature of a sample is changed throughout the measurement period [44]. Furthermore, there are three base measurements, namely, mass, temperature, and time, from which numerous additional measurements can be derived in the thermogravimetric analysis [45]. Consequently, taro mucilage was explored by TGA under a nitrogen atmosphere, and due to the presence of moisture in the mucilage sample, the initial weight loss was observed in the region of 40 to 110 °C. The highest weight loss occurred at temperatures between 230 and 310 °C, which corresponds to the structural degradation of mucilage. The temperature required for the mucilage to lose 50% of its weight is 293 °C, which is higher than the temperature required for okra mucilage to lose a similar amount of water, as reported by Sarkar et al. [38].

### 3.4. Rheological Properties of Taro Mucilage

Mucilage and other hydrocolloids are used in several industries (particularly in the food industry) because of their ability to modify the functional properties of foods. However, viscosity is one of the most important parameters of mucilage [46]. Viscosity can be affected by several factors, including shear rate, molecular weight, concentration, temperature, and salinity [22]. However, mucilage is considered a high-molecular-weight polymer. Several authors have proven that viscosity is directly influenced by the techno-functional properties and industrial applications (emulsifying properties, foaming capacity, and water-holding capacity) of taro mucilage. Moreover, viscosity can be responsible for the variation in the chemical composition (carbohydrate and amino acid profile). In this context, the rheological properties of taro mucilage were determined at 25 °C in a rheometer by Manhivi et al. [34]. To measure the steady-shear viscosity as a function of shear stress, the shear rate was measured. Taro mucilage showed Newtonian flow behavior at a low concentration (below 10%). On the other hand, the pseudo-plastic nature increased with an increase in concentration. Additionally, the consistency coefficient (k) revealed that viscosity was directly proportional to the concentration at any given shear rate. In another study, the viscosity of taro mucilage was evaluated by a rheometer equipped with a cone and plate geometry. Only a minor difference was seen between the curves of the different varieties of taro, which behave as power-law liquids to a very good approximation (r = 0.95–0.99) over this range of shear rates Njintang et al. [33].

## 4. Techno-Functional Property of Taro Mucilage 

Hydrocolloid polymers (high-molecular-weight biopolymers) are extensively used in the food and pharmaceutical industries. A hydroxyl group in a biopolymer causes viscous dispersions, which increases water attraction [16,47]. Hydrocolloids are also commonly used as stabilizers, thickening agents, dietary fiber, whipping agents, and fat substitutes. However, mucilage is water-soluble and often has remarkable functional properties, including water-holding capacity, oil-holding or binding capacity, and emulsifying, foaming, antioxidant, and antimicrobial properties [48]. Moreover, they also have diverse industrial applications in edible films and coatings, crystallization inhibition, and flavor encapsulation. High water-holding capacity is attributed to the presence of hydroxyl groups and protein substituents in the gum and mucilage structure [49]. The moist polymer or sample can hold water when exposed to an external centrifugal gravity force or compression. Water-holding capacity comprises the sum of linked water, physically trapped water, and hydrodynamic water, the latter of which contributes most to this capacity. Conversely, the inability to form a gel is due to the high solubility of mucilage, even at high concentrations, which results in a low water-holding capacity [50,51]. Furthermore, the elastic structure of mucilage is linked to good foaming properties. Foaming properties are influenced by a variety of parameters, including the presence of various chemicals in carbohydrates, structure, protein content, and molecular weight. The flexible structure of mucilage, which can minimize surface tension, is closely linked to its excellent foaming capacity [52].

## 5. Application of Taro Mucilage

Plant-derived additives or polymers can be used as natural thickeners or emulsifiers in the human diet and thus act as alternatives to synthetic polymers or additives [53]. Mucilage derived from plants can form a large number of network molecules due to their elasticity and thus can be widely used for edible films or edible coating in food packaging applications, thickening, encapsulation, inhibition of syneresis, control of crystallization, suspension of particulates, and stabilization of emulsions in the food industry, while it can act as disintegrators in tablets, tablet binders, and a variety of other pharmaceutical applications [54,55]. Additionally, mucilage is highly capable of swelling or solubilizing in aqueous systems, providing viscous material, similarly to the characteristics provided by fats. However, excessive intake of fats and oils can cause obesity, several types of cancer, cardiovascular diseases, and hypercholesterolemia [56]. Therefore, several authors have stated that mucilage should be considered as a potential ingredient in food industry applications, including fat replacers, emulsifiers, and dye removers [57].

### 5.1. Application of Mucilage as an Emulsifying Agent

**Figure 3:**
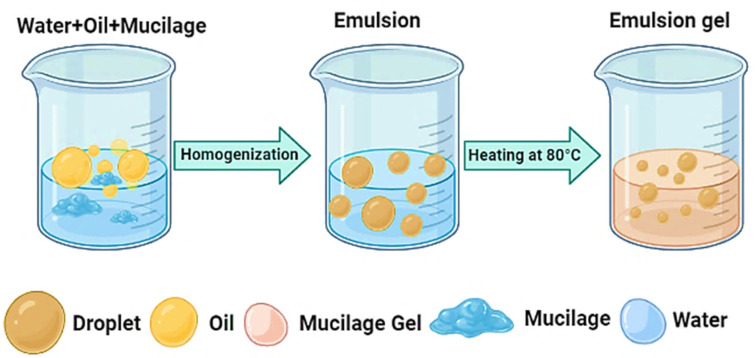
Proposed mechanism of emulsifying action of taro mucilage.

The emulsifying capacity of mucilage is an important function and has a wide range of applications in several industries. An emulsion is a homogeneous mixture of two immiscible liquids in which one phase is dispersed and distinct from the other [58]. Emulsifiers aid in the formation of fine dispersions (Figure 3), whereas stabilizers improve the stability of the dispersion of two or more ordinarily immiscible phases. A few mucilages and gums (e.g., gum arabic) can act as stabilizers and emulsifiers at the same time [59]. Furthermore, emulsifiers are amphiphilic compounds with a water-soluble polar component (hydrophilic) and a non-polar water-insoluble component (lipophilic or hydrophobic), and they are extensively employed in the food industry [60]. Monoglycerides, propylene glycol monoesters, lactylate esters, acetylated monoglycerides, and ethoxylated esters are the most common synthetic commercial emulsifiers. Natural emulsifiers include lecithin and gum arabic, as well as guar, xanthan, locust bean, and carrageenan gums. It can be utilized as a natural emulsifier in baking to produce sensory characteristics comparable to bread with additional commercial synthetic emulsifiers in the food industry [61]. However, it has been reported that taro mucilage has lower lipid content than other commercial emulsifiers [62]. In this context, the emulsifying properties of taro mucilage were evaluated by Andrande et al. [32]. The presence of a methyl group, which was observed in the infrared spectra, and the presence of low amounts of lipids may also contribute to the emulsifying power by providing a hydrophobic moiety. The hydrophilic portion of this emulsifier mainly consists of hydroxyl-containing carbohydrates. Therefore, it can be concluded that the protein content of taro mucilage, as well as weakly polar amino acids found in gums, is primarily responsible for its emulsifying capacity [63,64].

### 5.2. Application of Taro Mucilage as a Fat Replacer

**Figure 4:**
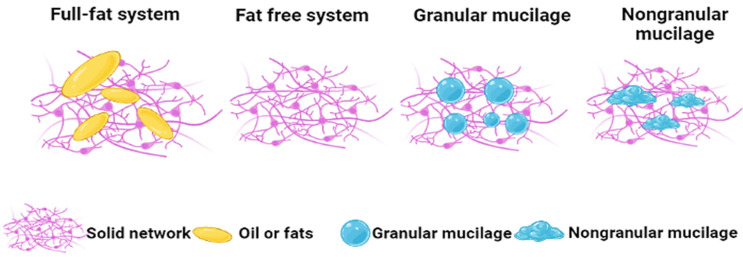
Application of taro mucilage as a fat replacer in bakery products.

In recent years, consumers have become more health-conscious. As a result, demand for low and reduced-fat foods is increasing, as customers need to change their food intake in order to lose weight [65]. Taro mucilage is an edible polysaccharide also known for its excellent functional properties (especially water-holding capacity) due to its several free hydroxyl groups and fiber-rich fractions, which may form bonds with water molecules [66]. Therefore, taro mucilage can mimic the properties of fats by increasing the viscosity of a system and promoting moisture retention in low-fat food products. This has a positive impact on the rheological aspects of foods (baked goods, meats, and dairy products) and is also correlated with softness and cooking loss [67]. Fat is important in baked products because it promotes dough lubrication and air incorporation and prevents gas-bubble coalescence during mixing. Moreover, the gelatinization of starch also reduces the presence of fat because the retardation of water is transferred to the starch granules [68]. In a study by Nagata et al. [69], physical, chemical, and sensory analyses of bread slices were carried out after the addition of taro mucilage. It was observed that bread containing taro mucilage showed excellent softness and sensorial properties. Additionally, it is believed that fat replacers have different mechanisms in common food products. In the case of bakery products, the interaction between fat and protein reduces the growth of the protein matrix during the process of dough formation, which is responsible for the short structures and softness of the bread, as shown in Figure 4. In this case, protein interaction with lipids can have an impact on the bread volume, the firmness of the crumbs, and the destabilization of the gas cell. Moreover, water activity can be reduced with the addition of mucilage to bakery products due to its good water-holding or absorbing properties [21]. In general, hydrogen bonding, steric interactions, electrostatic interactions, and covalent bonding are responsible for the techno-functional properties and applications of mucilage as fat replacers. The self-interactions of mucilage without competing with starchy polysaccharides and gluten proteins for available water in the system led to an increase in moisture content in some bakery products (e.g., eggless cake) [60].

### 5.3. Antioxidant Activity of Taro Mucilage

The hydroxyl radical is a potential oxidant that may react with all biological components, including proteins, lipids, and carbohydrates, and oxidative stress is known to have a role in a range of degenerative processes and diseases [70]. The damaging power of OH radicals is very strong, and the oxidation of fatty acids in biological membranes leads to the eventual destruction of the membrane, rearrangement of double bonds in unsaturated lipids, uptake of oxygen, and propagation of lipid radicals [71]. Therefore, research on antioxidants, especially plant-based polymer extracts, has become an important branch of science. Additionally, mucilage has a wide range of therapeutic properties, has become well known, and is widely used due to its ability to prevent oxidation [72]. The majority of plant-derived mucilages showed the ability to scavenge different free radicals produced and generated by various biological and chemical reactions. As a result, mucilage’s antioxidant function assists in the control of oxidative stress in the circulatory system [73]. Electron donor or hydrogen donor functional groups attached to polysaccharide chains provide polysaccharides with their antioxidant properties. These properties differ from one functional group to another and can be easily measured using in vitro antioxidant assays. However, very little literature is available on the antioxidant activity of taro mucilage [74]. For example, Nguimbou et al. [75] measured the antioxidant activity of mucilage from yellow and white giant swamp taro tubers. In this study, yellow taro mucilage showed higher antioxidant activity than white, while the chelating ability and the reducing power of taro increased with mucilage content. Moreover, the antioxidant activity of phenolic compounds is due to their redox characteristics, which allow them to act as singlet oxygen quenchers, hydrogen donors, and reducing agents. They also have metal-chelating potential. Therefore, they have excellent antioxidant properties [76].

### 5.4. Application of Taro Mucilage as Dye Remover

Dye wastewater primarily consists of residual dyes and auxiliary chemicals and can be one of the most significant contributors to water pollution in several industries, including textile effluent and food industries [77]. Over 50,000 tonnes of dye are discharged into wastewater each year, with the percentage varying depending on the dye type, fiber application, and degree of dye fixation. The proportion of dye that is lost to effluent varies among dye–fiber application systems [78]. Several methods for removing color from dyehouse effluent have been developed, with varying degrees of effectiveness, costs, and environmental impacts. However, it has been proven that color removal by sorbing dye molecules onto a substrate (adsorbent) can be a very successful and low-cost approach. Adsorption is used in numerous procedures that change or destroy the dye chromophore to remove color, but residual moieties are not always removed from the effluent and may cause environmental issues [79]. In addition, naturally, polysaccharides (gums, mucilage, and chitin) are superior to polymers for the adsorption of heavy metals and dyes due to the higher number of sites for possible chelation. Likewise, Mijinyawa et al. [41] proved that taro mucilage can remove methylene blue at basic pH under ultrasonication.

## 6. Health Attributes of Taro Mucilage

Taro is a traditional crop with significant nutritional and medicinal potential. The available literature indicates that different plant parts of taro contain a combination of bioactive chemicals, such as saponins, polyphenols, and alkaloids, and therapeutic properties, including hepatoprotective, immunoprotective, neuroprotective, anti-inflammatory, anti-hypertensive, anti-compulsive, and anti-carcinogenic properties [80]. The identification of several key bioactive components in mucilage has paved the way for its use as a treatment against various diseases. Since ancient times, it has been used to treat asthma, skin and neurological disorders, internal hemorrhaging, pneumonia, and hypertension [81]. Additionally, taro plays an effective protective role against cancer and cancer-related risk factors, including carcinogens and biological agents, and various pathophysiological conditions, such as inflammation and oxidative stress, while controlling metabolic dysfunctions and boosting the immune system. It is a promising potential alternative staple source with a lower glycemic index (GI) than potato; therefore, its consumption may reduce or prevent several diseases, including certain types of cancers [15]. Furthermore, taro is a gluten-free food that contains high calories, low fat, and low protein content. For this reason, its consumption can be beneficial for allergic and gluten-intolerant people. It is also an excellent source of vitamin B complexes, such as thiamin, pantothenic acid, riboflavin, folates, and pyridoxine [60]. In addition, taro rhizomes are rich in essential components of cells and body fluids, which can help to control blood pressure and also regulate heart diseases. Taro extracts containing tarin, which is one of the active chemicals, had an antimetastatic effect in mice after intraperitoneal injection of the highly metastatic murine breast cancer cell line 410.4, as studied by Kundu et al. [82]. Moreover, taro is a rich source of phenolic acids and flavonoids, which have excellent antioxidant properties due to the presence of the hydroxyl group in their structures. Flavonoids, the largest group of phenolic compounds found in various parts of the taro plant, have been associated with a lower risk of several degenerative diseases. The anti-inflammatory activity of taro mucilage is due to phenols and other antioxidants in taro that could curb the progression of cancer [83].

## 7. Conclusion, Future Research Perspective, and Challenges

Over the past years, plant-derived biopolymers (gums and mucilage) have received interest in food, pharma, and other industries due to their diverse industrial applications, including gelling, binding, emulsifying, and film coating capacities. These biopolymers have advantages over synthetic ones since they are easily available, biodegradable, cost-effective, and eco-friendly. Additionally, taro is a very important staple crop in the human diet and is mainly grown in the humid tropical regions of the world. It contains a high amount of mucilage, which is well known due to its diverse industrial applications and remarkable therapeutic and functional properties. Moreover, taro mucilage is extensively used as a fat replacer in bakery products, a dye remover, and an emulsifying agent. It also contains several carbohydrates and proteins that play a significant role in the improvement of the functional properties of foods. The majority of public and private agricultural funding for research is directed toward improving the production of high-value crops for high-paying markets or international trade. When taro is perceived as a “poor man’s crop” or “marginal crop,” this unfavorable attitude may deter the research needed to maximize several advantages of taro for small-scale farmers. Gaps in knowledge exist for numerous plants with significant potential value as local, regional, or global sources of food, medicine, fiber, and other uses. Moreover, research gaps on taro mucilage need to be addressed because of its potential use in several industrial applications as an effective eco-friendly, non-toxic, and cost-effective polymer. However, very few reports have been published on the utilization of mucilage in particular industrial applications.

## Figures and Tables

**Table 1:** Various extraction methods, yields, and chemical compositions of taro mucilage.

Extraction Method	Extraction Condition	Yield of Mucilage	Chemical Composition	References
Solvent treatment (ethyl alcohol), high- and low-temperature methods, filtration, and lyophilization	Mucilage was extracted at different five conditions (a) at room temperature, (b) at room temperature with ethanol precipitation, (c) at high temperature, (d) at high temperature with ethanol precipitation, and (e) at low temperature with ethanol precipitation	(a) 8.05% (b) 1.65% (c) 4.09% (d) 0.55% (e) 1.33%	Amino acids (proline, alanine, threonine, lysine, tyrosine, valine, phenylalanine, glycine, serine, arginine, and leucine)Monosaccharides (galactose, arabinose, and glucose)	[31]
Filtration and lyophilization	Filtrated mucilage was lyophilized for 72 h	9.63%	Amino acids (leucine, isoleucine, cysteine, tryptophan, and lysine) and monosaccharides (xylose, rhamnose, arabinose, fucose, mannose, galactose, fructose, and glucose.	[32]
Coldwater, filtration, and centrifugation	The filtrate was centrifuged at 13,000× *g* for 10 min at 4 °C	3.23%	Monosaccharides (arabinose, galactose, mannose, and rhamnose)	[37]
Coldwater, filtration, and centrifugation	The filtrate was centrifuged at 5000× *g* for 20 min at 4 °C	4.44%	Monosaccharides (glucose, galactose, arabinose, xylose, mannose, and glucuronic acid)	[34]
Solvent treatment	Six varieties were used to extract mucilage in a saline buffer containing 50 mM Tris pH 8, supplemented with solvents. The mixture was incubated overnight at 4 °C with agitation followed by centrifugation at 3000× *g* for 20 min.	3–18%	Sugars (arabinose, rhamnose, fucose, xylose, galacturonic acid, guluronic acid, mannose, galactose, and glucose)	[33]
High temperature	Mucilage was extracted by using two different solvents (a) methanol (b) acetone	9.4%1.2%	-	[38]

## Data Availability

Data sharing is not applicable to this article.
